# Identifying differentially expressed genes in goat mammary epithelial cells induced by overexpression of *SOCS3* gene using RNA sequencing

**DOI:** 10.3389/fvets.2024.1392152

**Published:** 2024-05-21

**Authors:** Ning Song, Cunxia Ma, Yuzhu Guo, Shuangshuang Cui, Shihao Chen, Zhi Chen, Yinghui Ling, Yunhai Zhang, Hongyu Liu

**Affiliations:** ^1^Anhui Province Key Laboratory of Local Livestock and Poultry Genetic Resource Conservation and Bio-breeding, College of Animal Science and Technology, Anhui Agricultural University, Hefei, China; ^2^Jiangsu Key Laboratory of Animal Genetic Breeding and Molecular Design, College of Animal Science and Technology, Yangzhou University, Yangzhou, China

**Keywords:** socs3, overexpression, lactation, lipid synthesis, goat

## Abstract

The suppressor of cytokine signaling 3 (SOCS3) is a key signaling molecule that regulates milk synthesis in dairy livestock. However, the molecular mechanism by which *SOCS3* regulates lipid synthesis in goat milk remains unclear. This study aimed to screen for key downstream genes associated with lipid synthesis regulated by *SOCS3* in goat mammary epithelial cells (GMECs) using RNA sequencing (RNA-seq). Goat *SOCS3* overexpression vector (PC-SOCS3) and negative control (PCDNA3.1) were transfected into GMECs. Total RNA from cells after *SOCS3* overexpression was used for RNA-seq, followed by differentially expressed gene (DEG) analysis, functional enrichment analysis, and network prediction. *SOCS3* overexpression significantly inhibited the synthesis of triacylglycerol, total cholesterol, non-esterified fatty acids, and accumulated lipid droplets. In total, 430 DEGs were identified, including 226 downregulated and 204 upregulated genes, following *SOCS3* overexpression. Functional annotation revealed that the DEGs were mainly associated with lipid metabolism, cell proliferation, and apoptosis. We found that the lipid synthesis-related genes, *STAT2 and FOXO6*, were downregulated. In addition, the proliferation-related genes *BCL2*, *MMP11*, and *MMP13* were upregulated, and the apoptosis-related gene *CD40* was downregulated. In conclusion, six DEGs were identified as key regulators of milk lipid synthesis following *SOCS3* overexpression in GMECs. Our results provide new candidate genes and insights into the molecular mechanisms involved in milk lipid synthesis regulated by *SOCS3* in goats.

## 1 Introduction

Ruminant milk is an important source of dietary nutrition that is rich in fatty acids for the human body. Compared to cow milk, the fat globules in goat milk are smaller in diameter and are easily digested by the human body, especially infants and children (1). Goat milk is rich in unsaturated and short- and medium-chain fatty acids, which have preventive and auxiliary therapeutic effects on coronary heart disease and diabetes (2). However, the functions of the key genes and the mechanisms of fatty acid synthesis in goat milk remain unclear. Therefore, discovering important molecules associated with lipid metabolism will contribute to improving the gene regulation network of milk fat synthesis and provide an experimental basis and new molecular targets for the improvement of milk quality.

The suppressor of cytokine signaling 3 (SOCS3) is a negative regulator that blocks multiple cellular signaling processes that can be activated by various cytokines, growth factors, and hormones. During the dry lactation period, low expression of *SOCS3* promotes mammary gland development and milk synthesis by enhancing prolactin signaling, suggesting that *SOCS3* participates in the regulation of lactation in dairy cows (3). SOCS3 negatively regulates Janus kinase 2 (JAK2)/signal transducer and activator of transcription 5 (STAT5) signaling pathway to inhibit milk synthesis and cell proliferation in bovine mammary epithelial cells (BMECs) (4). JAK2/STAT5, the mammalian target of rapamycin, and sterol regulatory element-binding protein (SREBP) signaling pathways regulate milk fat synthesis by inhibiting *SOCS3* expression in BMEC lines (5). In particular, n-3 and n-6 fatty acids inhibit *SOCS3* mRNA expression and downregulate nuclear factor-kappa B (NF-κB) and interleukin-6 levels in mice with high-fat diet-induced obesity (6). In addition, low STAT5 activity downregulates *SOCS3* expression and inhibits proliferation and lactation in BMECs (7).

Previous studies have shown that SOCS3 is closely associated with milk production in dairy livestock. However, the underlying mechanism through which *SOCS3* regulates milk lipid synthesis remains unclear. Therefore, in this study, we overexpressed *SOCS3* in goat mammary epithelial cells (GMECs) and screened for downstream molecules associated with milk lipid synthesis using RNA sequencing (RNA-seq). Our results provide a reference for follow-up studies on *SOCS3*-mediated key candidate genes and regulatory mechanisms of milk lipid synthesis in dairy livestock.

## 2 Materials and methods

### 2.1 Ethics statement

Wanlin white goats were obtained from Hefei Boda Animal Husbandry Science and Technology Development Co., Ltd. The animal study protocol was approved by the Animal Care and Use Committee of Anhui Agricultural University (SYXK 2016–007).

### 2.2 Isolation and culture of GMECs

Mammary gland tissue was collected from three Wanlin white goats (2–3 years old, second parturition) during early, peak, middle, late (15, 60, 120, and 210 days after parturition, respectively), and dry lactation (60 days before parturition) using a surgical method (8). Mammary tissues at different lactation stages were used for quantitative analysis of *SOCS3*. In addition, the primary GMECs were isolated from mammary tissue at the peak lactation period and then cultured using the tissue block method in saturated humidity at 37°C and 5% CO_2_. The GMECs were purified using a differential adhesion method to remove fibroblasts that adhered to the culture dishes faster than the GMECs. This process was performed for five passages to purify the GMECs for use in subsequent experiments. Cells were investigated using immunofluorescence with cytokeratin 18 (66187-1-Ig, Proteintech, Wuhan, China) and β-casein (bs-0466R, Bioss, Beijing, China; Supplementary Figure S1), which are specific to mammary epithelial cells. The cells were cultured in Dulbecco’s Modified Eagle Medium/Ham’s F-12 medium (SH30023, HyClone, Logan, UT, United States) containing 10% fetal bovine serum (11011–8,615, Tianhang, Hangzhou, China) and supplemented with 5 μg/mL bovine insulin (I8040, Solarbio, Beijing, China), 1 μg/mL hydrocortisone (H0888, Solarbio, Beijing, China), 10 ng/mL epidermal growth factor (PHG0311, Invitrogen, Carlsbad, CA, United States), and 100 U/mL penicillin/streptomycin (G8450, Solarbio, Beijing, China).

### 2.3 Construction of *SOCS3* overexpression vector

The coding region sequence of goat *SOCS3* (GenBank no. XM_018063683.1) was obtained from the National Center for Biotechnology Information database.^1^ The goat SOCS3 coding sequence was subcloned into a PCDN3.1 vector between the *BamHI* and *EcoRI* restriction enzyme sites to generate the PC-SOCS3 vector. The primer sequences (cleavage sites are underlined) used for PC-SOCS3 vector construction are as follows:

forward, *CCG*GAATTCATGGTCACCCACAGCAAGT;

reverse, *CGC*GGATCCCTAAAGCGGGGCATCGTACT.

### 2.4 Lipid synthesis assays

When GMECs had grown to 75% confluence, the overexpression vector PC-SOCS3 or negative control PCDNA3.1 was transfected into the GMECs using Lipofectamine 2000 (11,668,030, Invitrogen, Carlsbad, CA, United States). After transfection for 48 h, the cells were collected for Oil Red O staining (G1262, Solarbio, Beijing, China) and triacylglycerol (TAG, A110-1-1, Nanjing Jiancheng, Nanjing, China), total cholesterol (T-CHO, A111-1-1, Nanjing Jiancheng, Nanjing, China), and non-esterified fatty acid (NEFA, A042-2-1, Nanjing Jiancheng, Nanjing, China) assays.

### 2.5 Total RNA extraction and RNA-seq

After transfection for 48 h, total RNA from the *SOCS3* overexpression (*n* = 3) and control groups (*n* = 3) were extracted using a Total RNA Extraction Kit (R1200, Solarbio, Beijing, China), and the samples were preserved on dry ice (−78.5°C). Library construction and RNA-seq were performed by Novogene Science and Technology (Beijing, China). RNA quality and integrity were assessed using an RNA Nano 6,000 Assay Kit on a Bioanalyzer 2,100 system (Agilent Technologies, CA, United States). The RNA concentration was >300 ng/μL, and the RNA integrity number value was >9 (Supplementary Table S2). Library fragments were purified using the AMPure XP system (Beckman Coulter, Beverly, MA, United States) to select 370–420 bp-long cDNA fragments. The high-quality RNA samples were sequenced using an Illumina NovaSeq 6,000 system, generating 150-bp double-end reads. The sequencing depth was approximately 6.62 G, and the GC content was approximately 51.93% (Supplementary Table S3).

### 2.6 RNA-seq data analysis

Raw reads obtained by RNA-seq were filtered to remove low-quality reads and obtain clean ones. Clean paired-end reads were mapped to *Capra hircus* reference genome ARS1.2 (GCF001704415.2)^2^ using HISAT2 software. Gene expression was determined using the million mapped reads per kilobase value. R package (3.22.5) was used to calculate Pearson’s correlation coefficients for the samples. DESeq2 software (1.20.0) was used to analyze the differential expression between the control and *SOCS3* overexpression groups. The Benjamini–Hochberg algorithm was used to adjust the *p*-value (padj) to control for the false discovery rate. FoldChange >1.5 (| log2FoldChange | > 0.59) and adjusted *p*-value <0.05 were set as the significance threshold for differential expression. The Gene Ontology (GO) and Kyoto Encyclopedia of Genes and Genomes (KEGG) pathway enrichment analyses were performed using the ClusterProfiler R package (3.8.1), which corrected for gene length bias. Functional categories with an adjusted *p*-value <0.05 were considered statistically significant. Enrichment of GO terms and KEGG pathways with genes differentially expressed between treatments was analyzed based on the cumulative hypergeometric distribution.

### 2.7 RNA-seq data verification

Total RNA was extracted from the control and *SOCS3* overexpression groups, and reverse transcription was performed using the PrimeScript RT Kit (RR047, Takara, Shiga, Japan). Quantitative real-time PCR (qPCR) was performed on an Applied Biosystems StepOne Plus system (Thermo Scientific, Waltham, MA, United States) using a TB Green kit (RR820, Takara, Shiga, Japan). Ubiquitously expressed transcript (*UXT*) and ribosomal protein S9 (*RPS9*) were used as reference genes (9). The primers used for qPCR are listed in Supplementary Table S1. Data were normalized to internal controls and analyzed using the relative quantification (2^-ΔΔCt^) method. Data are shown as mean ± standard error and are statistically analyzed by an unpaired *t*-test using SPSS 20.0 software (IBM, Chicago, IL, United States). Differences were considered statistically significant for *p* < 0.05 (*p < 0.05, **p < 0.01).

## 3 Results

### 3.1 Milk lipid synthesis assay

**Figure 1:**
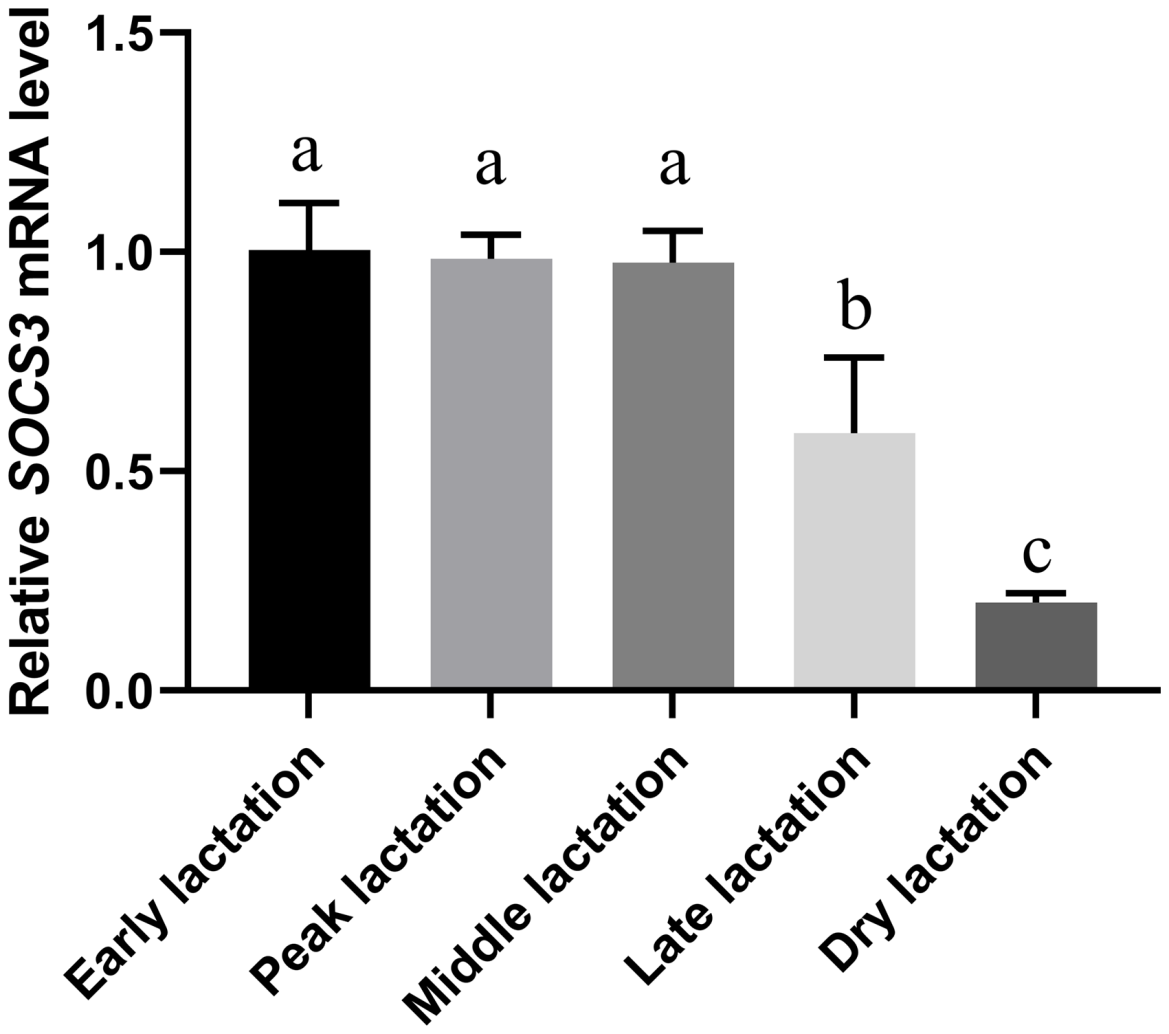
Suppressor of cytokine signaling 3 (*SOCS3*) gene expression in goat mammary gland of different lactation stages. Mammary gland samples were obtained from Wanlin white goats during early, peak, middle, late, and dry lactation stages. Data were normalized to the early lactation stage. Different lowercase letters represent significant differences in *SOCS3* levels (*p* < 0.05).

The quantitative results from goat mammary tissue showed that the abundance of *SOCS3* mRNA was highest in the early and peak lactation periods and lowest in the dry lactation period (Figure 1). *SOCS3* gene expression decreased globally from the early to dry lactation periods.

**Figure 2:**
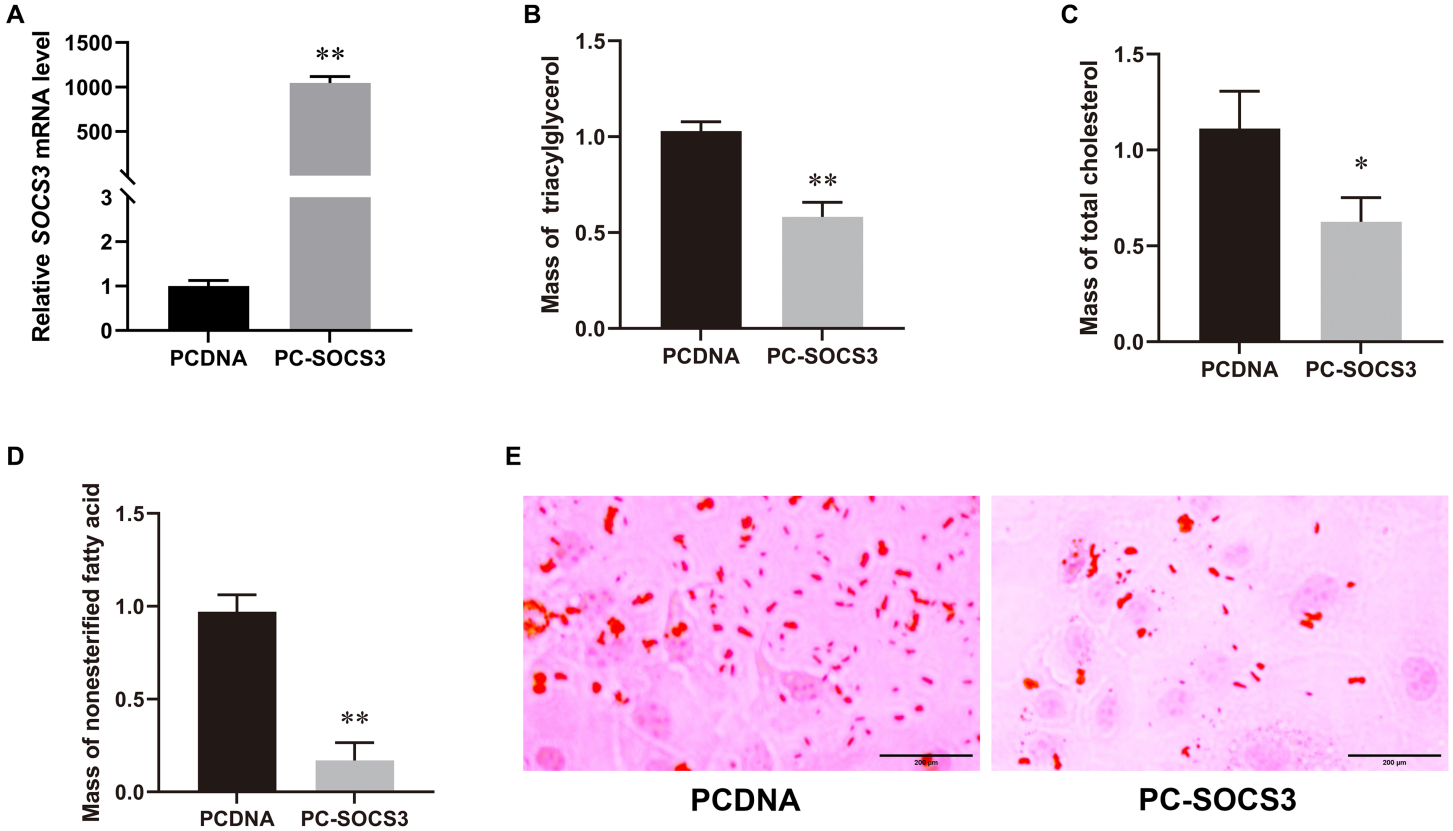
*SOCS3* overexpression inhibits lipid synthesis in goat mammary epithelial cells (GMECs). **(A)** Overexpression efficiency of *SOCS3* gene. **(B)** Effect of *SOCS3* overexpression on triacylglycerols. **(C)** Effect of *SOCS3* overexpression on total cholesterol. **(D)** Effect of *SOCS3* overexpression on non-esterified fatty acids. **(E)** Effect of *SOCS3* overexpression on lipid droplets determined using Oil Red O staining.

Transfection efficiency was detected by qPCR, and the expression of *SOCS3* in the overexpression group was 1,050-fold higher than that in the control group (Figure 2A). To understand the effect of *SOCS3* on milk lipid synthesis, we quantified the levels of TAG, T-CHO, NEFA, and lipid droplets. We found that the levels of TAG, T-CHO, NEFA, and lipid droplets decreased in the *SOCS3* overexpression group (Figures 2B–E). Therefore, *SOCS3* inhibits milk lipid synthesis in GMECs.

### 3.2 Evaluation of RNA-seq data quality

**Figure 3:**
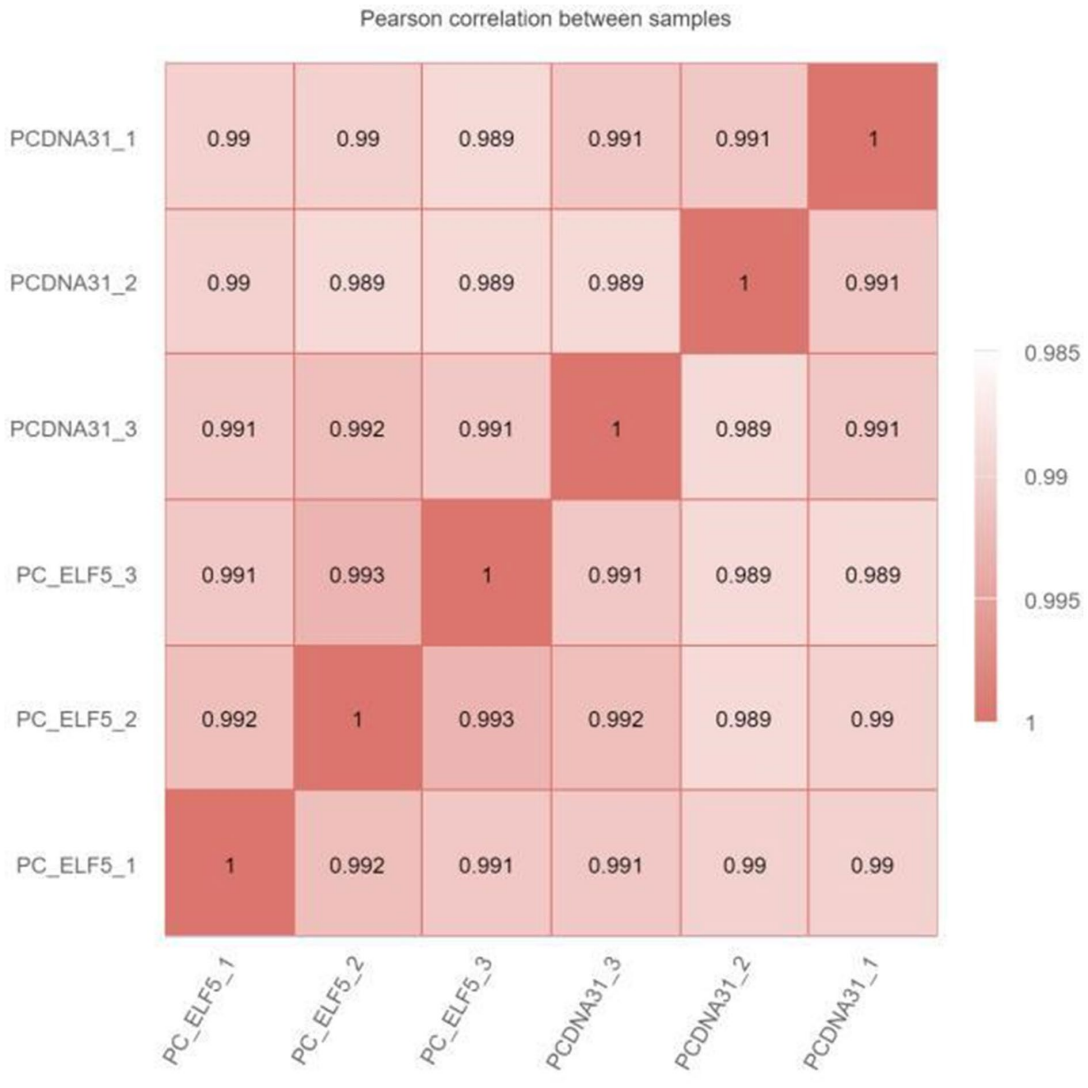
Heat map of the correlation coefficient between RNA sequencing (RNA-seq) samples. The numbers in the box represent the correlation coefficient of the two samples.

The RNA-seq was used to explore the regulatory network of SOCS3 that affects milk lipid synthesis. When the six samples were grouped, the correlation coefficient (R^2^) between the *SOCS3* overexpression and control groups was approximately 0.99, indicating that the three biological samples in each group showed similarly high performance (Supplementary Figure S2; Figure 3). After filtering the raw reads with adapter sequences and low-quality reads, a total average of 44,091,933 clean reads were obtained with phred base quality scores of Q20 > 97% and Q30 > 94% (Supplementary Table S3).

### 3.3 Differentially expressed gene analysis

**Figure 4:**
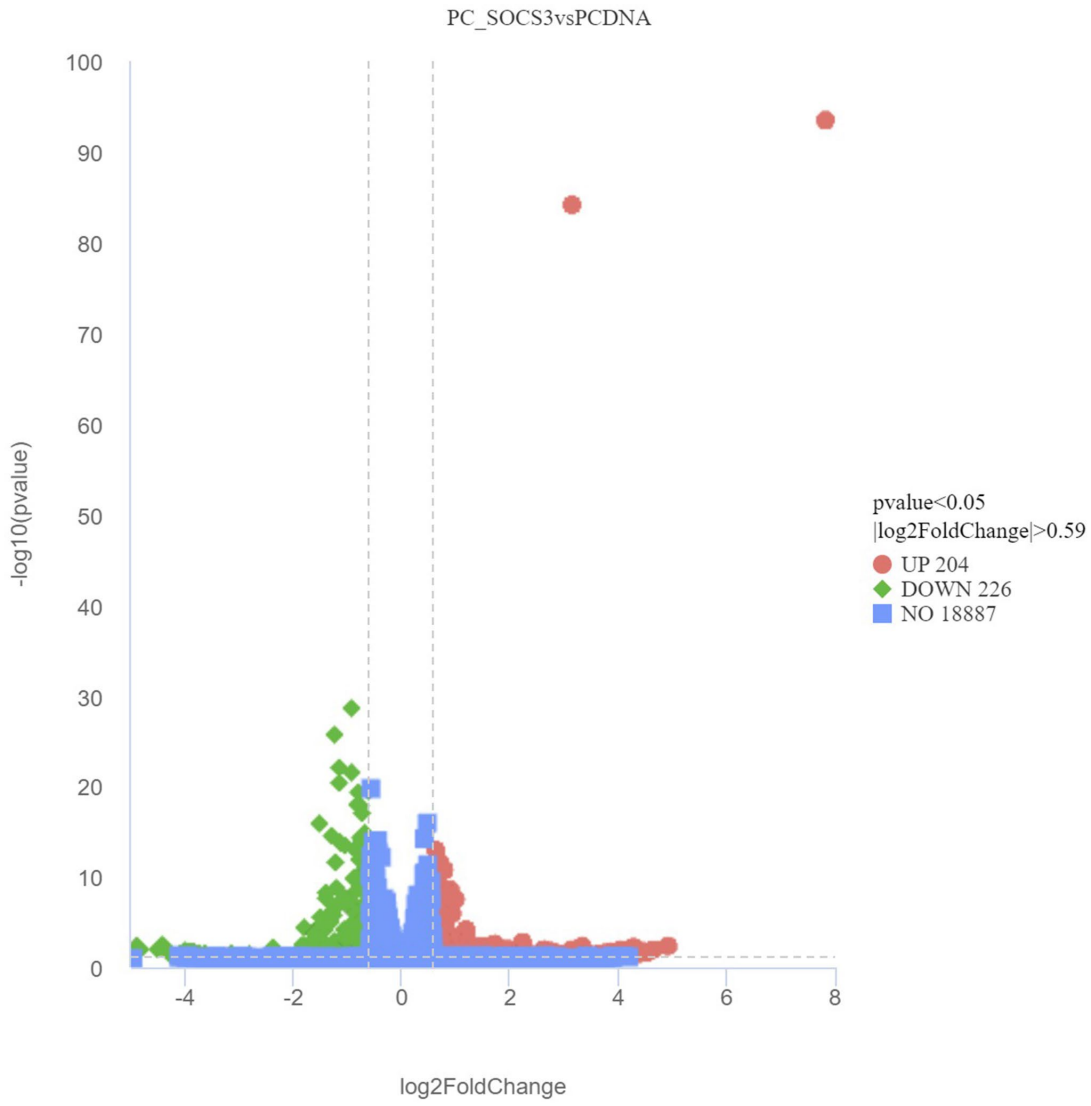
Volcano plot of differentially expressed genes (DEGs) between the PCDNA and PC-SOCS3 groups. The red dots represent significant upward adjustments, the green dots represent significant downward revisions, and the gray dots indicate no difference. Filter criteria: Adjusted *p*-value <0.05 and FoldChange >1.5 (| log2FoldChange | > 0.59). Genes in the middle of the two dotted lines and below the horizontal dotted line are not DEGs.

While verifying the difference in gene expression due to *SOCS3* overexpression in GMECs, 430 DEGs were identified, including 204 upregulated and 226 downregulated genes. Volcano plots of DEGs between the PCDNA3.1 and PC-SOCS3 groups are shown in Figure 4. A heatmap of the hierarchical clustering analysis of DEGs between the two groups is shown in Supplementary Figure S3. Lipid synthesis is closely associated with lipid metabolism, cell proliferation, and apoptosis. Among the DEGs, downregulated genes, such as *STAT2*, forkhead box O6 (*FOXO6*), and *CD40,* were associated with lipid synthesis and cell apoptosis, whereas upregulated genes, such as matrix metalloproteinase 9 (*MMP9*), *MMP11*, and B-cell lymphoma 2 (*BCL2*), were associated with cell growth.

### 3.4 Validation and dynamic expression of DEGs

**Figure 5:**
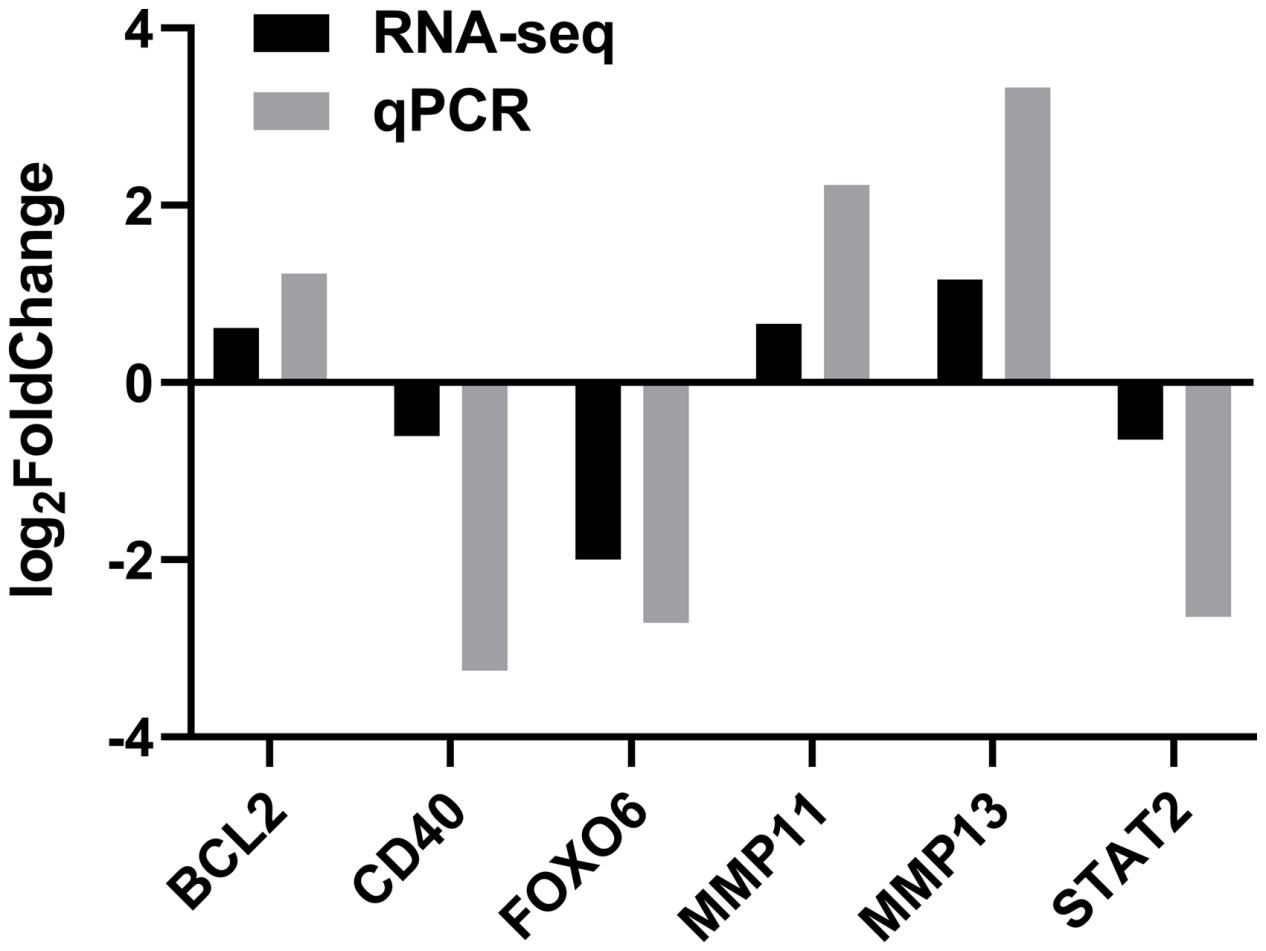
Validation of DEGs by quantitative real-time PCR (qPCR) in the *SOCS3* overexpression group.

To verify the DEGs involved in lipid synthesis, cell proliferation, and apoptosis and the RNA-seq results, we selected six DEGs, comprising *STAT2, FOXO6, CD40, MMP11*, *MMP13*, and *BCL2,* for validating the RNA-seq data by qPCR. The qPCR data corroborated the RNA-seq findings, indicating the reliability of the RNA-seq data (Figure 5).

### 3.5 Functional annotation of DEGs

**Figure 6:**
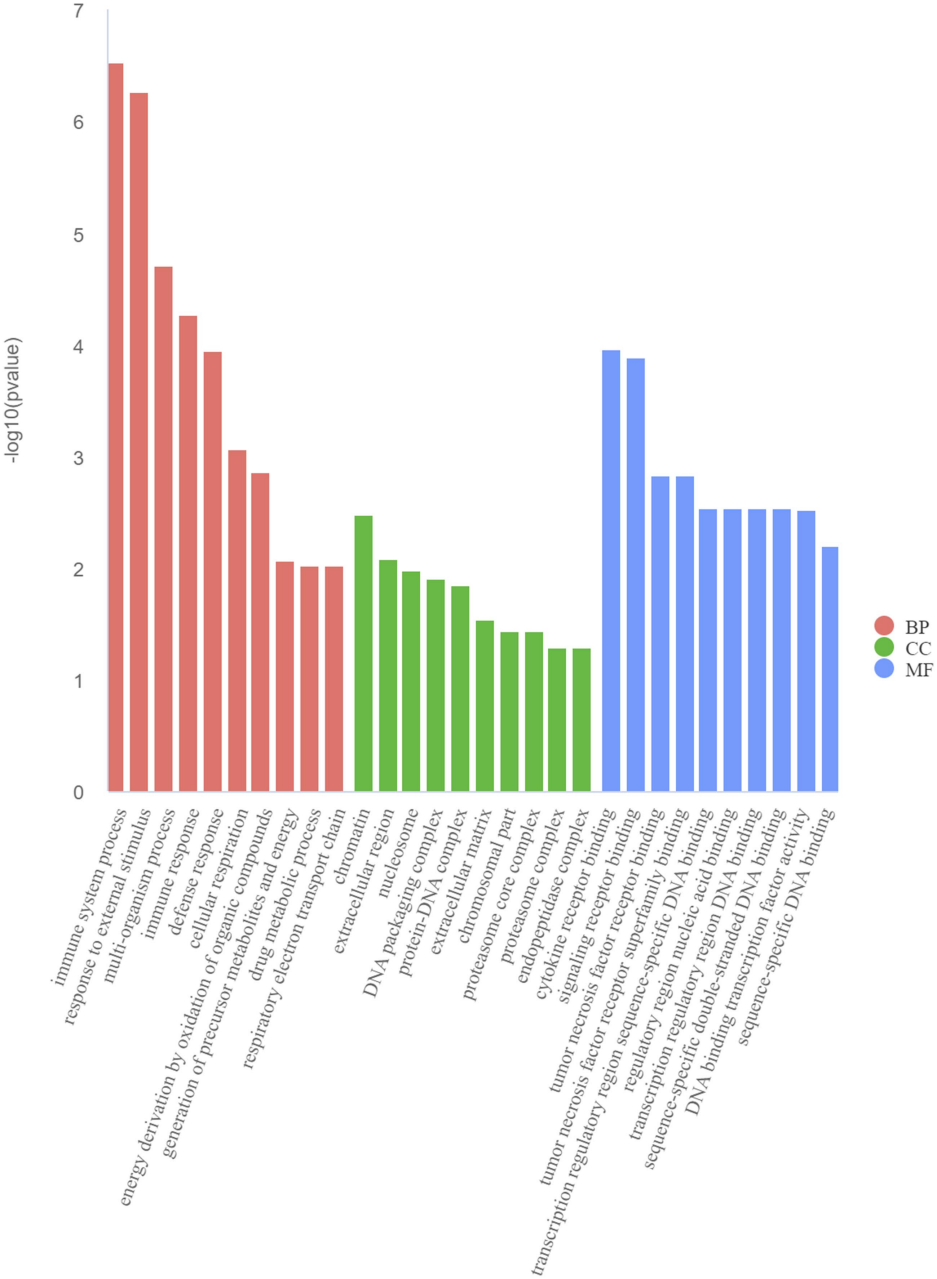
Top 10 Gene Ontology (GO) terms of DEGs determined using the GO analysis. BP (biological processes), CC (cell components), and MF (molecular functions).

**Figure 7:**
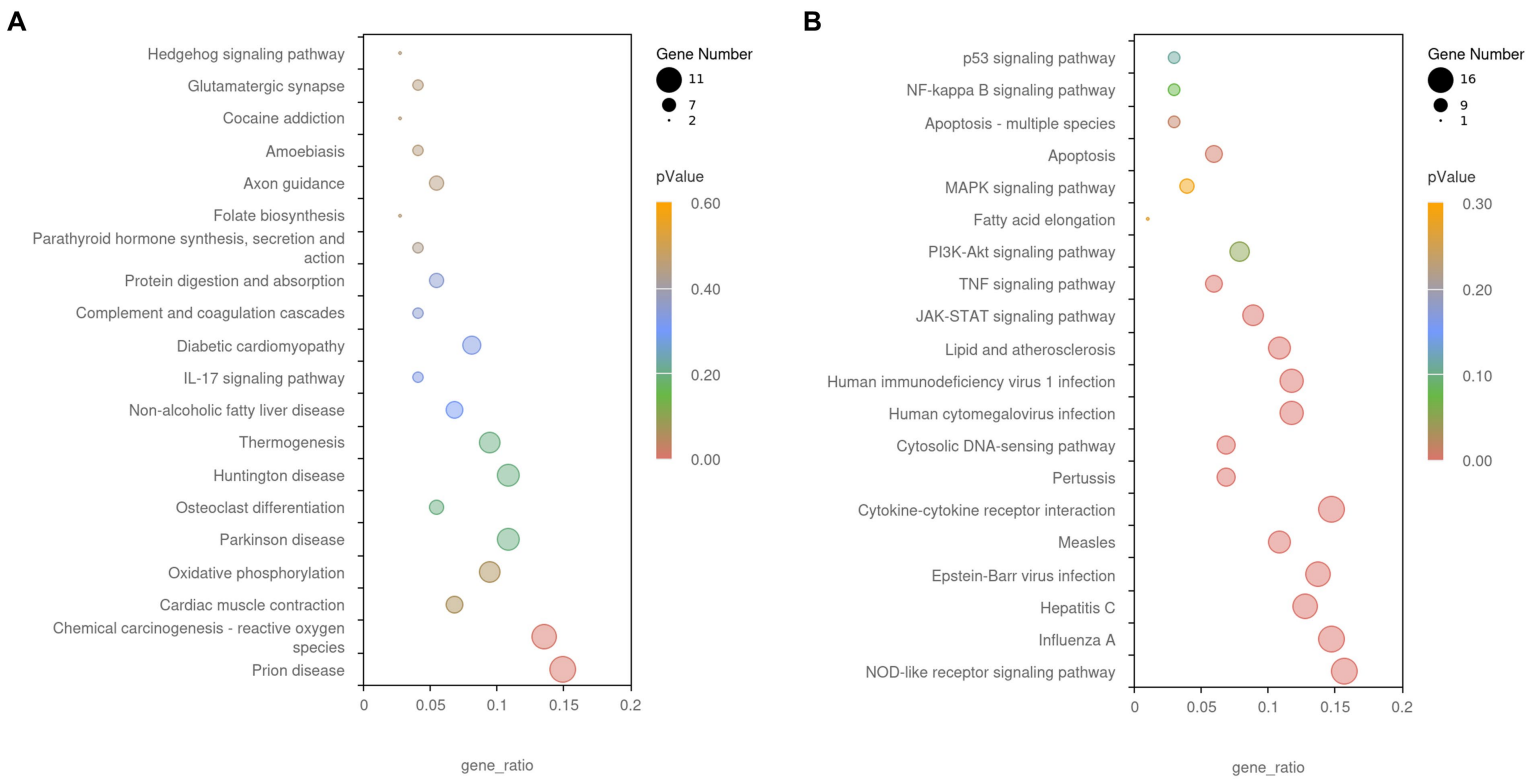
The Kyoto Encyclopedia of Genes and Genomes (KEGG) enrichment analysis of DEGs. **(A)** Upregulated gene enrichment pathways. **(B)** Downregulated gene enrichment pathways. Adjusted *p*-value <0.05 indicates the significance of pathway enrichment.

**Figure 8:**
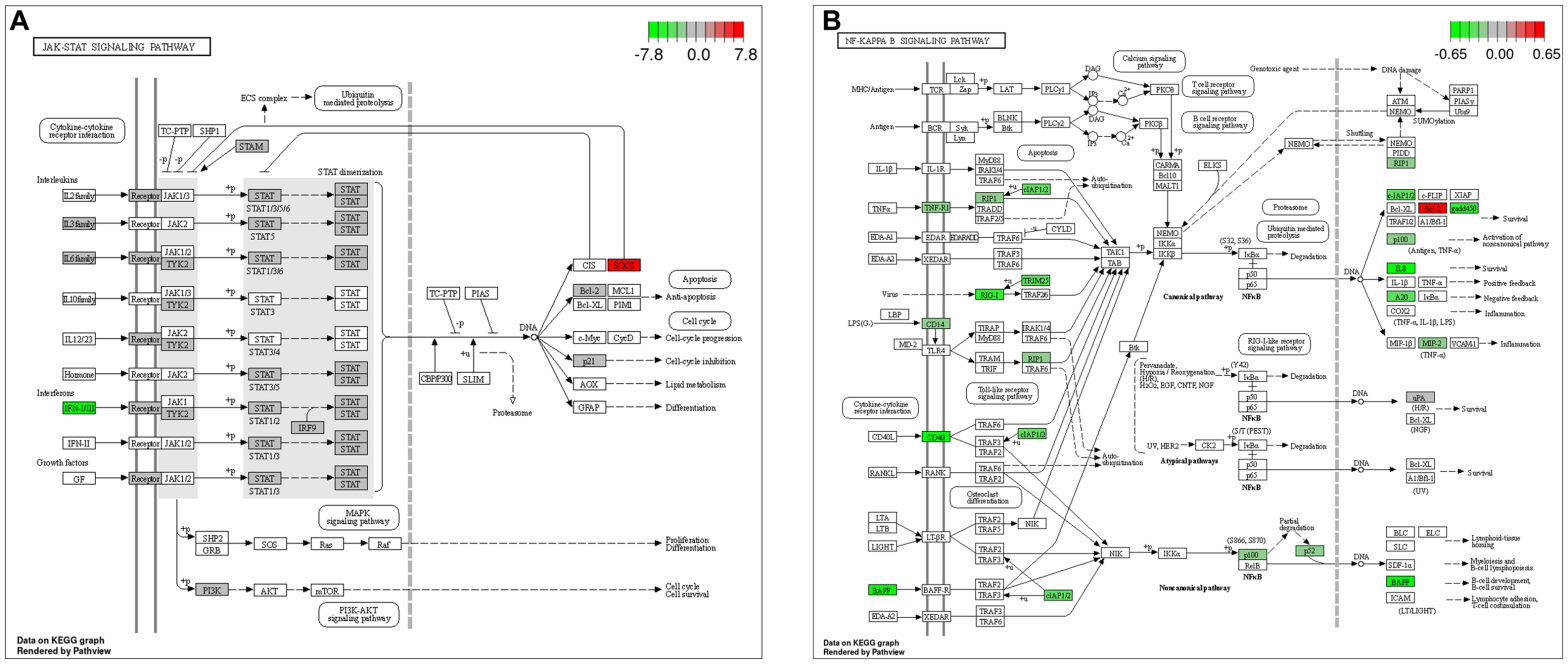
Gene network analysis of DEGs. **(A)** Janus kinase (JAK)/signal transducer and activator of transcription (STAT) signaling pathway. **(B)** Nuclear factor-kappa B (NF-κB) signaling pathway.

The GO and KEGG enrichment analyses of DEGs were performed. The GO functional annotation was performed to identify the biological functions of the DEGs. A total of 430 DEGs were enriched in 404 GO terms, including 190 in biological process terms, 45 in cellular component terms, and 169 in molecular function terms (Figure 6). Pathway analysis of the DEGs associated with lipid synthesis was performed using the KEGG database. The upregulated genes were mainly enriched in chemical carcinogenesis-reactive oxygen species and prion disease, whereas the downregulated genes were enriched in Epstein–Barr virus infection, influenza A, nucleotide-binding oligomerization domain (NOD)-like receptor, and tumor necrosis factor (TNF) signaling pathways (Figure 7). Gene network analysis showed that multiple DEGs were enriched in the JAK/STAT and NF-κB signaling pathways, which were closely associated with lipid metabolism, cell proliferation, and apoptosis progress (Figure 8).

## 4 Discussion

Milk lipid synthesis in dairy livestock is regulated by various signaling molecules (10). As a negative regulator of signal transduction, SOCS3 affects milk lipid metabolism in ruminants (11). In this study, *SOCS3* overexpression decreased milk lipid synthesis in GMECs. We then explored *SOCS3* regulation of potential downstream signaling molecules involved in milk lipid synthesis by RNA-seq. In BMECs, SOCS3 binds to the phosphorylated tyrosine residues of JAK2 and inhibits milk lipid metabolism by blocking STAT5 activity (12). SOCS3 can inhibit the binding of leptin and its receptor during feedback, resulting in pathway blockage of the signal transduction of leptin, insulin, and growth hormone signals in mice (13). SOCS3 disrupts fatty acid synthesis by reducing SREBP activity, thus validating its involvement in milk lipid synthesis in mammary epithelial cells. In this study, the GO and KEGG analyses showed that several DEGs were enriched in lipid metabolism and cell survival following *SOCS3* overexpression.

STAT2 plays a critical role in immune responses to extracellular and intracellular stimuli and upregulates the expression and secretion of pro-inflammatory mediators (14). In particular, STAT2 promotes the transcription and expression of acetyl-CoA carboxylase, thus enhancing lipid synthesis in colorectal cancer cells (15). Fatty acid binding protein 4 upregulates macrophage inflammation-related interleukin-6 (IL-6) level and regulates lipid metabolism in atherosclerosis induced by activation of the JAK2/STAT2 pathway (16). Unphosphorylated STAT2 bridges the interferon-stimulated response and NF-κB elements in the *IL-6* promoter and increases cancer cell survival by enhancing IL-6 expression (17). SOCS3 and SOCS1 lead to sustained cytokine activation of STAT2 and contribute to the progression of inflammation in mouse macrophages (18). SOCS3 is a negative regulator of the JAK/STAT pathway and is a cytokine-inducible SH2 domain-containing protein involved in cell proliferation, differentiation, apoptosis, and immune regulation (19). In this study, *SOCS3* overexpression significantly inhibited lipid synthesis, which was speculated to be mediated by *STAT2* downregulation.

FOXO6 is a transcription factor that mediates insulin signaling during glucose and lipid metabolism. FOXO6 constitutes a distinct route by which the liver orchestrates insulin-dependent regulation of gluconeogenesis and triglyceride-rich very low-density lipoprotein production. Increased hepatic FOXO6 activity activates glucose production and triglyceride secretion in response to fasting (20). Phosphorylated FOXO6 promotes resistance to oxidative stress and inhibition of pro-inflammatory mediators by facilitating the nuclear translocation of NF-κB induced by lipopolysaccharides in aged rats (21). FOXO6 activation induces hepatic lipid accumulation by increasing the expression and transcriptional activity of peroxisome proliferator-activated receptor gamma during endoplasmic reticulum stress in HepG2 cells (22). Furthermore, it triggers cellular triglyceride-mediated lipid accumulation in the livers of aged rats fed a high-fat diet, leading to hepatic steatosis and hyperglycemia. Conversely, the expression of genes involved in lipid synthesis is reduced in FOXO6-knockout mice, resulting in decreased hepatic lipid accumulation (23). FOXO6 promotes inflammation by activating cytokine IL-1β and induces lipid triglyceride accumulation in the mouse liver and human hepatocellular carcinomas (24). In our study, *SOCS3* overexpression significantly inhibited the accumulation of triglycerides, which may be associated with *FOXO6* downregulation.

Survival affects lactation and lipid metabolism in mammary epithelial cells. In this study, *SOCS3* overexpression significantly upregulated the mRNA levels of *BCL2*, *MMP11,* and *MMP13* and downregulated the mRNA levels of *CD40*, which are involved in cell proliferation and apoptosis. As an inhibitor of apoptosis, the loss of BCL2 affects lipid metabolism, and BCL2 probably has a close relationship with lipid peroxidation and reactive oxygen species production (25). Furthermore, *BCL2* is a downstream gene of the phosphatidylinositol-4,5-bisphosphate 3-kinase (PI3K)/protein kinase B (AKT) signaling pathway, which is involved in the cell cycle, metabolism, and apoptosis. In our study, KEGG enrichment analysis showed that the PI3K/AKT pathway was downregulated, which was consistent with the expression trend of *BCL2*. Activation of the PI3K/AKT pathway enhances cell proliferation in mice and GMECs (26). The PI3K/AKT pathway regulates milk lipid metabolism, cell proliferation, and fatty acid synthesis in GMECs (27). *MMP11* overexpression in macrophages promotes the proliferation and migration of breast cancer cells and monocyte recruitment (28). MMP13 contributes to the proliferation, migration, and anchorage-independent clonogenicity of mouse mammary tumorigenic cell lines (29). The tumor necrosis factor receptor family member CD40 is critical for the immune system and induces tumor cell-specific apoptosis (30). However, how the regulation of *BCL2*, *MMP11*, *MMP13*, *and CD40* mediated by *SOCS3* overexpression affects lipid metabolism and the complex relationship between cell survival and lipid metabolism remains unclear.

Interestingly, functional enrichment analysis showed that *SOCS3* overexpression downregulated multiple apoptosis-related biological processes, such as the TNF- and NOD-like receptor signaling pathways. The TNF signaling pathway includes signal transducers and adaptor proteins. A transcriptome study showed that Fumonisin B1 induces apoptosis via the TNF signaling pathway in porcine kidney cells (31). Excessive inflammatory overload-associated activation of the TNF pathway ultimately induces hepatic lipid accumulation in high-fat diet-fed mice (32). NOD-like receptor X1 regulates multiple cellular processes, including antiviral immunity, apoptosis, reactive oxygen species generation, and mitochondrial metabolism (33). Mitochondrial NOD-like receptors control apoptosis in response to intrinsic and extrinsic cues in murine embryonic fibroblasts and human embryonic kidney 293 cells (34). However, whether the regulation of lipid metabolism by *SOCS3* is associated with the TNF- and NOD-like receptor pathways needs to be further explored.

## 5 Conclusion

Our study identified 430 DEGs, including 226 downregulated and 204 upregulated genes, by overexpressing *SOCS3* in GMECs. Among these, *STAT2*, *FOXO6, BCL2, MMP11, MMP13*, and *CD40* may be key regulatory genes involved in milk lipid metabolism. These genes play crucial roles in lipid synthesis, cell proliferation, and apoptosis. These results provide a reference for follow-up studies on the functions of key candidate genes involved in milk lipid synthesis regulated by *SOCS3* in goats.

## Data availability statement

The datasets presented in this study can be found in online repositories. The names of the repository/repositories and accession number(s) can be found at: https://www.ncbi.nlm.nih.gov/, PRJNA1080325.

## Ethics statement

The animal studies were approved by Animal Care and Use Committee of Anhui Agricultural University. The studies were conducted in accordance with the local legislation and institutional requirements. Written informed consent was obtained from the owners for the participation of their animals in this study.

## Author contributions

NS: Conceptualization, Data curation, Investigation, Methodology, Software, Validation, Writing – original draft. CM: Conceptualization, Data curation, Investigation, Methodology, Software, Validation, Writing – original draft. YG: Data curation, Investigation, Methodology, Writing – original draft. SCu: Data curation, Investigation, Methodology, Writing – original draft. SCh: Formal analysis, Software, Writing – review & editing. ZC: Formal analysis, Software, Writing – review & editing. YL: Validation, Writing – review & editing. YZ: Conceptualization, Resources, Supervision, Writing – review & editing. HL: Conceptualization, Resources, Supervision, Writing – review & editing.

## Footnotes

^1^ https://www.ncbi.nlm.nih.gov/

^2^https://www.ncbi.nlm.nih.gov/

## References

[1] Prosser CG. Compositional and functional characteristics of goat milk and relevance as a base for infant formula. J Food Sci. (2021) 86:257–65. doi: 10.1111/1750-3841.15574, PMID: 33438254

[2] Clark S Garcia MBM. A 100-year review: advances in goat milk research. J Dairy Sci. (2017) 100:10026–44. doi: 10.3168/jds.2017-13287, PMID: 29153153

[3] Wall EH Auchtung-Montgomery TL Dahl GE McFadden TB. Short communication: short-day photoperiod during the dry period decreases expression of suppressors of cytokine signaling in mammary gland of dairy cows. J Dairy Sci. (2005) 88:3145–8. doi: 10.3168/jds.S0022-0302(05)72997-0, PMID: 16107404

[4] Huang Y Zhao F Luo C Zhang X Si Y Sun Z. Socs3-mediated blockade reveals major contribution of jak2/stat5 signaling pathway to lactation and proliferation of dairy cow mammary epithelial cells in vitro. Molecules. (2013) 18:12987–3002. doi: 10.3390/molecules181012987, PMID: 24141248 PMC6270101

[5] Zong JX Shen JL Liu XL Liu JY Zhang J Zhou CH. Lithium chloride promotes milk protein and fat synthesis in bovine mammary epithelial cells via hif-1α and β-catenin signaling pathways. Biological Trace Element Res. (2023) 201:180–95. doi: 10.1007/s12011-022-03131-8, PMID: 35080710

[6] Tung Y Chen Y Fan T Fong T Chiu W. Effects of dietary adjustment of n-3:n-6 fatty-acid ratio to 1:2 on anti-inflammatory and insulin-signaling pathways in ovariectomized mice with high fat diet-induced obesity. Heliyon. (2023) 9:e20451. doi: 10.1016/j.heliyon.2023.e20451, PMID: 37817999 PMC10560786

[7] Jiao B Zhang X Wang S Wang L Luo Z Zhao H. Microrna-221 regulates proliferation of bovine mammary gland epithelial cells by targeting the stat5a and irs1 genes. J Dairy Sci. (2019) 102:426–35. doi: 10.3168/jds.2018-15108, PMID: 30366615

[8] Song N Luo J Huang L Tian H Chen Y He Q. Mir-204-5p and mir-211 synergistically downregulate the alpha(s1)-casein content and contribute to the lower allergy of goat milk. J Agric Food Chem. (2021) 69:5353–62. doi: 10.1021/acs.jafc.1c01147 33939400

[9] Song N Chen Y Luo J Huang L Tian H Li C. Negative regulation of alpha(s1)-casein (csn1s1) improves beta-casein content and reduces allergy potential in goat milk. J Dairy Sci. (2020) 103:9561–72. doi: 10.3168/jds.2020-18595, PMID: 32828499

[10] Singh A Malla WA Kumar A Jain A Thakur MS Khare V. Review: genetic background of milk fatty acid synthesis in bovines. Trop Anim Health Prod. (2023) 55:328. doi: 10.1007/s11250-023-03754-6, PMID: 37749432

[11] Khan MZ Khan A Xiao JX Ma YL Ma JY Gao J. Role of the jak-stat pathway in bovine mastitis and milk production. Animals (Basel). (2020) 10:2107. doi: 10.3390/ani10112107, PMID: 33202860 PMC7697124

[12] Geng Z Shan X Lian S Wang J Wu R. Lps-induced socs3 antagonizes the jak2-stat5 pathway and inhibits beta-casein synthesis in bovine mammary epithelial cells. Life Sci. (2021) 278:119547. doi: 10.1016/j.lfs.2021.119547, PMID: 33930363

[13] Yin YL Liu WW Dai YL. Socs3 and its role in associated diseases. Hum Immunol. (2015) 76:775–80. doi: 10.1016/j.humimm.2015.09.037 26429311

[14] Lee C An H Cho ES Kang HC Lee JY Lee HS. Stat2 stability regulation: an intersection between immunity and carcinogenesis. Exp Mol Med. (2020) 52:1526–36. doi: 10.1038/s12276-020-00506-6, PMID: 32973222 PMC8080578

[15] Yang Y Luo D Shao Y Shan Z Liu Q Weng J. Circcaprin1 interacts with stat2 to promote tumor progression and lipid synthesis via upregulating acc1 expression in colorectal cancer. Cancer Commun (Lond). (2023) 43:100–22. doi: 10.1002/cac2.12380, PMID: 36328987 PMC9859733

[16] Xu L Zhang H Wang Y Yang A Dong X Gu L. Fabp4 activates the jak2/stat2 pathway via rap1a in the homocysteine-induced macrophage inflammatory response in apoe−/− mice atherosclerosis. Lab Investig. (2022) 102:25–37. doi: 10.1038/s41374-021-00679-2, PMID: 34725437 PMC8695379

[17] Nan J Wang Y Yang J Stark GR. Irf9 and unphosphorylated stat2 cooperate with nf-κb to drive il6 expression. Proc Natl Acad Sci USA. (2018) 115:3906–11. doi: 10.1073/pnas.1714102115, PMID: 29581268 PMC5899435

[18] Duncan SA Sahu R Dixit S Singh SR Dennis VA. Suppressors of cytokine signaling (socs)1 and socs3 proteins are mediators of interleukin-10 modulation of inflammatory responses induced by chlamydia muridarum and its major outer membrane protein (momp) in mouse j774 macrophages. Mediat Inflamm. (2020) 2020:7461742. doi: 10.1155/2020/7461742, PMID: 32684836 PMC7333066

[19] Sobah ML Liongue C Ward AC. Socs proteins in immunity, inflammatory diseases, and immune-related cancer. Front Med (Lausanne). (2021) 8:727987. doi: 10.3389/fmed.2021.727987, PMID: 34604264 PMC8481645

[20] Lee S Dong HH. Foxo integration of insulin signaling with glucose and lipid metabolism. J Endocrinol. (2017) 233:R67–79. doi: 10.1530/JOE-17-0002, PMID: 28213398 PMC5480241

[21] Kim DH Park MH Chung KW Kim MJ Park D Lee B. Suppression of foxo6 by lipopolysaccharide in aged rat liver. Onco Targets Ther. (2015) 6:34143–57. doi: 10.18632/oncotarget.6219, PMID: 26506521 PMC4741442

[22] Kim DH Kim BM Chung KW Choi YJ Yu BP Chung HY. Interaction between chop and foxo6 promotes hepatic lipid accumulation. Liver Int. (2020) 40:2706–18. doi: 10.1111/liv.14594, PMID: 32639626 PMC7689817

[23] Kim DH Lee S Noh SG Lee J Chung HY. Foxo6-mediated apoc3 upregulation promotes hepatic steatosis and hyperlipidemia in aged rats fed a high-fat diet. Aging. (2024) 16:4095–115. doi: 10.18632/aging.205610, PMID: 38441531 PMC10968681

[24] Kim ME Lee JS Kim TW Park MH Kim DH. Foxo6-mediated txnip induces lipid accumulation in the liver through nlrp3 inflammasome activation. Endocrinol Metab (Seoul). (2024) 39:127–39. doi: 10.3803/EnM.2023.1826, PMID: 38417829 PMC10901662

[25] Liu Y Zhang HS Fan C Liu FY Li SY Li JJ. Potential role of bcl2 in lipid metabolism and synaptic dysfunction of age-related hearing loss. Neurobiol Dis. (2023) 187:106320. doi: 10.1016/j.nbd.2023.106320, PMID: 37813166

[26] Xu LY Chen JM Jia LT Chen X Moumin FA Cai JT. Slc1a3 promotes gastric cancer progression via the pi3k/akt signalling pathway. J Cell Mol Med. (2020) 24:14392–404. doi: 10.1111/jcmm.16060, PMID: 33145952 PMC7753768

[27] Chen K Hou J Song Y Zhang X Liu Y Zhang G. Chi-mir-3031 regulates beta-casein via the pi3k/akt-mtor signaling pathway in goat mammary epithelial cells (gmecs). BMC Vet Res. (2018) 14:369. doi: 10.1186/s12917-018-1695-6, PMID: 30482199 PMC6258393

[28] Kang SU Cho SY Jeong H Han J Chae HY Yang H. Matrix metalloproteinase 11 (mmp11) in macrophages promotes the migration of her2-positive breast cancer cells and monocyte recruitment through ccl2-ccr2 signaling. Lab Investig. (2022) 102:376–90. doi: 10.1038/s41374-021-00699-y, PMID: 34775491

[29] Dumortier M Ladam F Damour I Vacher S Bieche I Marchand N. Etv4 transcription factor and mmp13 metalloprotease are interplaying actors of breast tumorigenesis. Breast Cancer Res. (2018) 20:73. doi: 10.1186/s13058-018-0992-0, PMID: 29996935 PMC6042225

[30] Ibraheem K Yhmed AMA Nasef MM Georgopoulos NT. Traf3/p38-jnk signalling crosstalk with intracellular-trail/caspase-10-induced apoptosis accelerates ros-driven cancer cell-specific death by cd40. Cells. (2022) 11:3274. doi: 10.3390/cells11203274, PMID: 36291141 PMC9600997

[31] Chen J Yang SH Huang S Yan R Wang MY Chen S. Transcriptome study reveals apoptosis of porcine kidney cells induced by fumonisin b1 via tnf signalling pathway. Food Chem Toxicol. (2020) 139:111274. doi: 10.1016/j.fct.2020.111274, PMID: 32198028

[32] Xu MX Sun Y Dai XL Zhan JX Long TT Xiong MX. Fisetin attenuates high fat diet-triggered hepatic lipid accumulation: a mechanism involving liver inflammation overload associated tace/tnf-α pathway. J Funct Foods. (2019) 53:7–21. doi: 10.1016/j.jff.2018.12.007

[33] Schweizer L Bi PY Killackey SA Girardin SE. Nlrx1: versatile functions of a mitochondrial nlr protein that controls mitophagy. Biom J. (2024) 47:100635. doi: 10.1016/j.bj.2023.100635, PMID: 37574163 PMC10837482

[34] Killackey SA Rahman MA Soares F Zhang AB Abdel-Nour M Philpott DJ. The mitochondrial nod-like receptor nlrx1 modifies apoptosis through sarm1. Mol Cell Biochem. (2019) 453:187–96. doi: 10.1007/s11010-018-3444-3, PMID: 30191480

