# Supplementary material for: Identifying differentially expressed genes in goat mammary epithelial cells induced by overexpression of SOCS3 gene using RNA sequencing
Source: Front Vet Sci. 2024 May 21;11:1392152. doi: 10.3389/fvets.2024.1392152 (PMC11148363; doi:10.3389/fvets.2024.1392152)
Supplement: Supplementary file 1 [file Table_1.DOCX]

**Supplementary Table S1.** Primer sequences used for quantitative real-time PCR

| NCBI accession | gene | primer sequence (5′-3′) | length (bp) |
| --- | --- | --- | --- |
| XM_018063683.1 | *SOCS3* | F, CCAAAGACTTCGGTTCGGGAC | 154 |
|  |  | R, CGGCTGGATTCTTGTGCTTG |  |
| NM_001314213.1 | *BCL2* | F, AGAGCGTCAACCGGGAGATG | 167 |
|  |  | R, CAGCCAGGAGAAATCAAACAGG |  |
| XM_005688619.3 | *CD40* | F, TGCCGTCCTGTTGGTATCTG | 85 |
|  |  | R, CATAGGGTGCAGGGCCTTAG |  |
| XM_013962879 | *FOXO6* | F, GAGGGCGCCGAGGACT | 289 |
|  |  | R, TGTGCCGGATGGAGTTCTTC |  |
| XM_018060912.1 | *MMP11* | F, GTGATCGACTTCACCAGGTACT | 112 |
|  |  | R, AGTGGACATCCCCTTCTCGG |  |
| XM_005689359.3 | *MMP13* | F, TCTTGTTGCTGCCCATGAGT | 202 |
|  |  | R, ACATTTGTCCGGCGTTTTCG |  |
| XM_018048069.1 | *STAT2* | F, TGGGGAAGCGTTTGGCTGAA | 239 |
|  |  | R, CATTTGGGGTCTGAGCCTGGTT |  |
| XM_005700842.2 | *UXT* | F, CAGCTGGCCAAATACCTTCAA | 125 |
|  |  | R, GTGTCTGGGACCACTGTGTCAA |  |
| XM_005709411.1 | *RPS9* | F, CCTCGACCAAGAGCTGAAG | 64 |
|  |  | R, CCTCCAGACCTCACGTTTGTTC |  |

**Supplementary Table S2.** Quality inspection results of RNA samples

| Sample name | Nucleic acid number | Concentration (ng/µL) | RIN value |
| --- | --- | --- | --- |
| PCDNA3.1_1 | FKRN230049083-1A | 340.00 | 9.50 |
| PCDNA3.1_2 | FKRN230049084-1A | 426.00 | 9.60 |
| PCDNA3.1_3 | FKRN230049085-1A | 415.00 | 9.60 |
| PC_SOCS3_1 | FKRN230049086-1A | 310.00 | 9.70 |
| PC_SOCS3_2 | FKRN230049087-1A | 446.00 | 9.80 |
| PC_SOCS3_3 | FKRN230049088-1A | 467.00 | 9.70 |

Note: The RNA integrity number (RIN) value indicates the parameter of RNA integrity, and a value > 7.0 indicates good ntegrity.

**Supplementary Table S3.** Sequencing results of RNA samples

| Sample name | Clean reads | Clean bases | Q20 | Q30 | GC content | Mapping rate |
| --- | --- | --- | --- | --- | --- | --- |
| PCDNA3.1_1 | 45652310 | 6.85G | 97.84 | 93.86 | 51.54 | 96.16 |
| PCDNA3.1_2 | 42846022 | 6.43G | 98.11 | 94.6 | 51.96 | 96.34 |
| PCDNA3.1_3 | 43974376 | 6.6G | 98.12 | 94.61 | 51.59 | 96.65 |
| PC_SOCS3_1 | 44891756 | 6.73G | 97.99 | 94.27 | 51.95 | 96.37 |
| PC_SOCS3_2 | 44205816 | 6.63G | 97.74 | 93.65 | 52.13 | 95.6 |
| PC_SOCS3_3 | 42981320 | 6.45G | 97.87 | 93.96 | 52.42 | 95.77 |

Note: Q20 (the proportion of bases with a phred base quality score of Q20; the proportion of read bases whose error rate was less than 1%), Q30 (the proportion of bases with a phred base quality score of Q30; the proportion of read bases whose error rate was less than 0.1%) and GC content (the proportion of G and C base numbers of the total bases).


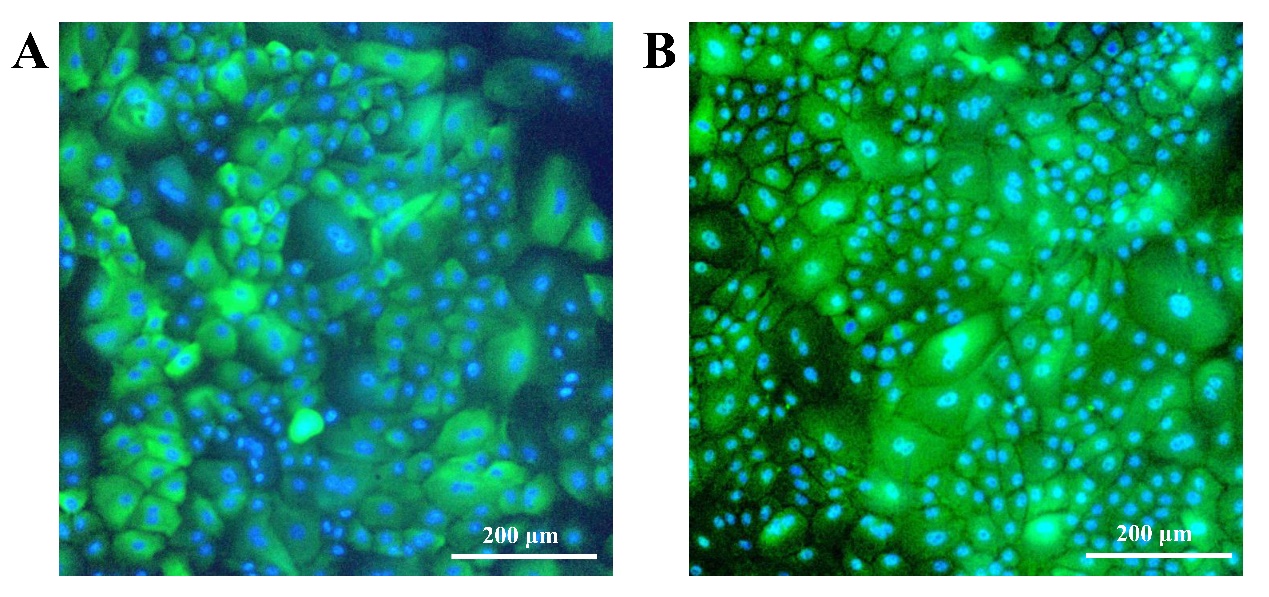


**Supplementary Figure S1.** Goat mammary epithelial cells were investigated by immunofluorescence with Cytokeratin 18 (A) and β-casein (B).


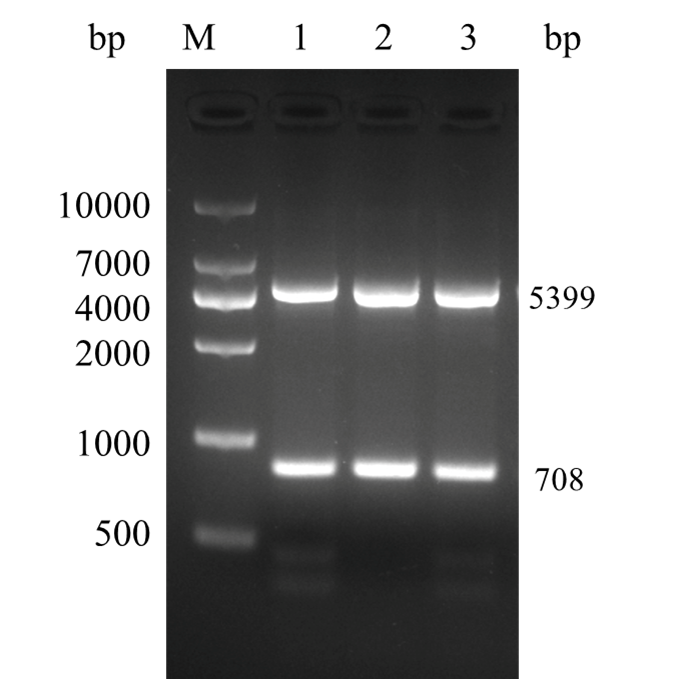


**Supplementary Figure S2.** Identification of the suppressor of cytokine signaling 3 (*SOCS3*) gene overexpression vector by double enzyme digests.


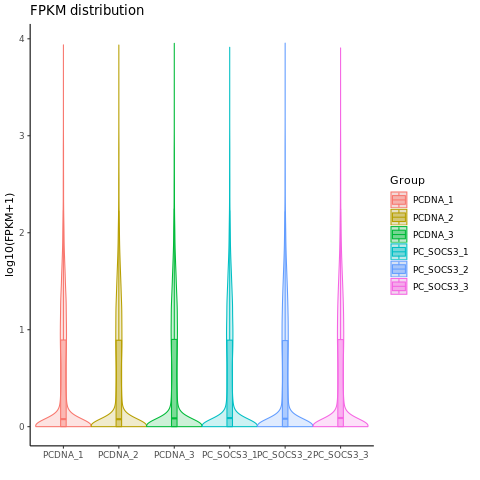
 **Supplementary Figure S3.** Statistical chart of gene number in different expression level intervals by RNA sequencing (RNA-seq).


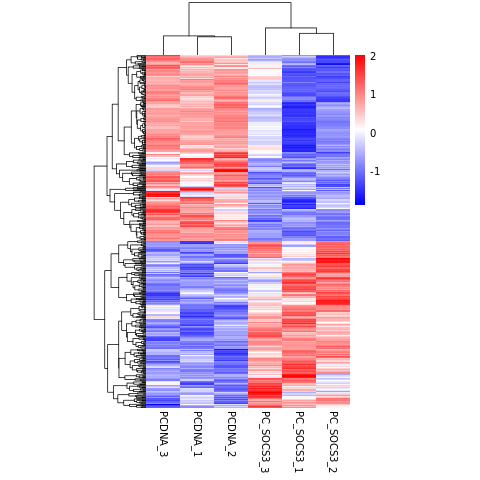


**Supplementary Figure S4.** Stratified clustering of differentially expressed genes (DEGs) between PC-SOCS3 and PCDNA heat map. The abscissa is the sample name, and the ordinate is the normalized million mapped reads per kilobase value of the difference genes. Red represents upward adjustment and blue represents downward adjustment; The darker the color means the higher/lower the expression level.
